# Triglycerides and Open Angle Glaucoma – A Meta-analysis with meta-regression

**DOI:** 10.1038/s41598-017-08295-1

**Published:** 2017-08-10

**Authors:** Laura Pertl, Georg Mossböck, Andreas Wedrich, Martin Weger, Oliver Königsbrügge, Günther Silbernagel, Florian Posch

**Affiliations:** 1 Department of Ophthalmology, Medical University of Graz, Auenbruggerplatz 4, 8036 Graz, Austria; 2 Clinical Division of Haematology and Haemostaseology, Department of Medicine I, Medical University of Vienna, Währinger Gürtel 18-20, 1090 Vienna, Austria; 3 Division of Angiology, Department of Internal Medicine, Medical University of Graz, Auenbruggerplatz 15, 8036 Graz, Austria; 4 Division of Oncology, Department of Internal Medicine, Medical University of Graz, Auenbruggerplatz 15, 8036 Graz, Austria

## Abstract

Although intraocular pressure is the main the risk factor for the development of glaucoma, other risk factors such as vascular dysfunction might play an additional pathogenic role. Hypertriglyceridemia, which may lead to vascular dysfunction, has been implicated in the development of glaucoma. The objective of this meta-analysis was to investigate the association of triglyceride levels with the risk of glaucoma in case-control studies. Seventeen case-control studies were included investigating the difference in triglyceride levels in patients with glaucoma (N = 1 391) compared to subjects without glaucoma (N = 25 575). In random effects meta-analysis, the pooled mean triglyceride level across all studies and patients with and without glaucoma was 132.9 mg/dL (95%CI: 124.0–141.7). Patients with glaucoma had significantly higher mean triglyceride levels than patients without glaucoma (absolute difference = 14.2 mg/dL, 95%CI: 5.8–22.5, p < 0.0001). A considerable amount of heterogeneity of included studies was observed (I^2^ = 66.2%, heterogeneity χ^2^ = 47.4 on 16 degrees of freedom, p < 0.0001). In conclusion, this meta-analysis of case-control studies found that patients with glaucoma had higher mean triglyceride levels than patients without glaucoma. This finding is consistent with the concept that hypertriglyceridemia represents an additional risk factor for glaucoma. Whether this association is causal and/or might be modified by glaucoma medications remains to be investigated.

## Introduction

Elevated intra-ocular pressure (IOP) is the main risk factor for the development of glaucoma. However, many patients develop glaucoma despite an IOP in the normal range, and not all patients with elevated IOP develop glaucoma^1^. Therefore, other risk factors have been implicated in the pathogenesis of glaucoma including structural abnormalities and functional dysregulation of the vasculature supplying the optic nerve and the surrounding retinal tissue^2^. The presence of vascular dysfunction in glaucoma patients was recently postulated in a large clinical study, the Leuven Eye Study^3^.

Hypertriglyceridaemia is often seen in patients with metabolic syndrome and type 2 diabetes^4^. Based on recent genetic studies, triglycerides are increasingly considered to be a causal cardiovascular risk factor^5–7^. Thus, novel therapies to reduce circulating triglycerides are currently being developed^8^. The role of triglycerides in the pathogenesis and progression of glaucoma remains unclear. Several case-control studies report a significant association of triglycerides with glaucoma^9, 10^ and with intraocular pressure^11, 12^. However, this was not confirmed in all studies^13, 14^ and a recent study by Ko *et al*.^15^ even found a significant inverse relationship between high triglyceride levels and glaucoma. The objective of this meta-analysis was to investigate the association of triglyceride levels with the risk of glaucoma in case-control studies.

## Results

### Study characteristics

Seventeen studies were included investigating the difference in triglyceride levels in patients with glaucoma (number of patients (N) 1 391) compared to patients without glaucoma (N 25 575) (Table 1).

**Table 1:** Triglyceride summary statistics of the 17 included studies.

Study	Glaucoma	N_p_^a^	N_c_^b^	Triglycerides_p_	Triglycerides_c_	p-value^c^	TotalChol_p_^d^	TotalChol_c_	LDL_p_^e^	LDL_c_	HDL_p_^f^	HDL_c_
Yilmaz^37^	NTG	33	40	145	121	0.02	221.5	191	138.3	123.2	47.1	42.8
	PEX	31		132			209.4		132.5		46.4	
Ogurel^20^	PEX	19	16	192.1	260.7	0.44	191.2	212.8	107.1	119.6	47.5	42.8
			35	223.6			202.7		119.7		48.9	
Shim^38^	NTG	55	92	174.9	141	0.51	164.8	161.1				
	POAG	20		191.6			180.7					
Modrzejewska^39^	POAG	56	54	110.4	97.9	0.1	200.2	165.2	133.1	83.2	38.3	44.1
Kurtul^21^	PEX	20	52	145	140	0.92	220	217.39	150	138	47	44
			47		143			205		127		47
Bossuyt^24^	NTG	30	33	94	98	0.72	201	215	111	120	69	75
Zhao^19^	Glaucoma	191	2835	154	145.6	0.52					55.4	55.5
Kim#2^40^	NTG	300	17940	128	120.2	0.37					54.3	53
Kim#1^14^	OAG	80	4015	112.2	112.3	0.79	178	176.5			46.6	50
Davar^18^	POAG	40	40	165.9	99.5	<0.01	211.2	162.3				
Lee^41^	NTG	45	35	170.4	148	0.34	203.8	207.9	120.7	124.6	59.1	60
Yuki^4^ ^2^	NTG	43	40	146.6	128.9	0.34	224.5	216				
Engin^43^	Glaucoma	160	31	170.5	118	N/d^g^	201.2	173				
Pavljasevic^16^	POAG	50	50	210.6	180.5	Ns^h^	237	230	152.9	157.5	56	54
Choi^44^	NTG	38	38	114.8	108.1	0.68	167.5	181	107.9	118.3	53.1	54.9
Chiba^17^	NTG	31	61	100	86	Ns	204	214				
	POAG	20		105			210					
Elisaf^13^	POAG	49	72	145.3	152.7	Ns	233.7	237.5	160	156	50.6	51.8

^a^N_p_: Number of patients with glaucoma.

^b^N_c_: Number of patients without glaucoma.

^c^p-value: P-Value regarding the difference in triglyceride levels.

^d^TotalChol: Total cholesterol levels.

^e^LDL: Low density lipoprotein levels.

^f^HDL: High density lipoprotein levels.

^g^N/d: Not defined.

^h^Ns: Not significant.

The largest study included 300 patients with normal tension glaucoma (NTG) and 17940 patients without glaucoma, while the smallest study included 19 patients with pseudoexfoliation glaucoma (PEX glaucoma) and 51 patients without glaucoma. Five studies included patients with primary open angle glaucoma (POAG), five studies patients with NTG, two studies patients with PEX glaucoma, two studies patients with NTG and POAG, one study patients with NTG and PEX glaucoma and two studies included all glaucoma types. Patients without glaucoma were hospital controls in six studies, community controls in four studies and not defined in seven studies (Table 2).

**Table 2:** Characteristics of the 17 included studies.

Study	Patients			Controls			Inclusion of				Triglycerides as primary Endpoint
	Number	Glaucoma Type	Definition	Number	Definition	Hospital-based	Diabetics	Smokers	Cardio-vascular disease	Lipid-lowering medication	
Yilmaz^37^	32	NTG	≤21 mmHg + glaucomatous changes in the optic disc and visual field	40	21mmHg + NO glaucomatous changes in optic disc and visual field	N/d^a^	Yes	No	Yes	N/d	Yes
	31	PEX-Glaucoma	Presence of pseudoexfoliation material + > 21 mmHg + glaucomatous changes in the optic disc and visual field								
Ogurel^20^	19	PEX-Glaucoma	Presence of pseudoexfoliation material + > 21 mmHg	16	Presence of pseudo-exfoliation material + ≤ 21 mmHg	N/d	No	No	N/d	N/d	No
				35	NO presence of pseudoexfoliation material + ≤ 21 mmHg						
Shim^38^	55	NTG	≤21 mmHg + glaucomatous changes in the optic disc and visual field + open-angle on gonioscopy	92	N/d	Yes	ONLY Diabetics	N/d	N/d	N/d	No
	20	POAG	>21 mmHg + glaucomatous changes in the optic disc and visual field + open-angle on gonioscopy								
Modrze-jewska^39^	56	POAG	glaucomatous changes in the optic disc, visual field and scanning laser polarimeter	54	N/d	N/d	No				
	No	No	No	Yes							
Kurtul^21^	20	PEX-Glaucoma	Presence of pseudoexfoliation material + > 21 mmHg + glaucomatous changes in the optic disc and visual field + open-angle on gonioscopy	52	Presence of pseudo-exfoliation material + < 21 mmHg	N/d	Yes	N/d	Yes	No	Yes
				47	>21 mmHg + NO glaucomatous changes in the optic disc and visual field						
Bossuyt^24^	30	NTG	≤21 mmHg + glaucomatous changes in the optic disc and visual field	33	N/d	No	No	N/d	No	Yes	No
Zhao^19^	191	Glaucoma	Self-reported	2835	Self-reported	No	Yes	Yes	N/d	N/d	No
Kim #2^40^	300	NTG	≤21 mmHg + glaucomatous changes in the optic disc and visual field + open-angle	17940	N/d	No	Yes	No	N/d	N/d	Yes
Kim #1^14^ (risk factors)	80	OAG	>21 mmHg or glaucomatous changes in the optic disc or optic disc haemorrhage or presence of retinal nerve fibre layer defect	4015	≤21 mmHg + NO glaucomatous changes in optic disc + open angle + absence of disc haemorrhage + absence of retinal nerve fibre layer defect + optic disc satisfying the ISNT rule	No	Yes	N/d	N/d	N/d	Yes
Davari, 2014	40	POAG	N/d	40	N/d	Yes	No	No	No	No	Yes
Lee^41^	45	NTG	≤21 mmHg + glaucomatous changes in the optic disc and visual field + open anterior chamber by gonioscopy	35	N/d	Yes	No	N/d	No	No	No
Yuki^42^	43	NTG	≤21 mmHg + glaucomatous changes in the optic disc and/or presence of nerve fibre layer defect + open anterior chamber by gonioscopy	40	≤21 mmHg + NO glaucomatous changes in the optic disc	Yes	No	No	No	N/d	No
Engin^43^	160	Glaucoma	>21 mmHg and/or glaucomatous changes in the optic disc and/or visual field	31	N/d	N/d	N/d	N/d	N/d	N/d	No
Pavljasevic^16^	50	POAG	N/d	50	N/d	Yes	N/d	N/d	N/d	N/d	Yes
Choi^44^	38	NTG	≤21 mmHg + glaucomatous changes in the optic disc and visual field + open anterior chamber by gonioscopy	38	≤21 mmHg + NO glaucomatous changes in the optic disc and visual field + NO disc haemorrhage, notching, excavation or asymmetry of the vertical cup to disc ratio > 0.2	Yes^b^	No	N/d	No	No	Yes
Chiba^17^	31	NTG	N/d	61	N/d	N/d	No	Yes	No	N/d	No
	20	POAG	N/d								
Elisaf^13^	49	POAG	Glaucomatous changes in the optic disc and visual field + open anterior chamber by gonioscopy	72	Normal intraocular pressure	N/d	Yes	Yes	No	No	Yes

^a^N/d: Not defined. ^b^Control patients were recruited from medical staff and clinic patients.

### Triglyceride levels in patients with and without glaucoma

In random-effects meta-analysis of all 17 included studies, the mean triglyceride level pooled across studies and patients with and without glaucoma was 132.9 mg/dL (95%CI: 124.0–141.7, Table 1, Supplementary Figure 1). The mean triglyceride level strongly varied between studies (I^2^ = 91.9%, heterogeneity χ^2^ = 197.1 on 16 degrees of freedom, p < 0.0001), and ranged from 93.3 mg/dL in the study by Chiba *et al*. to 223.5 mg/dL in the study by Ogurel *et al*.

**Figure 1:**
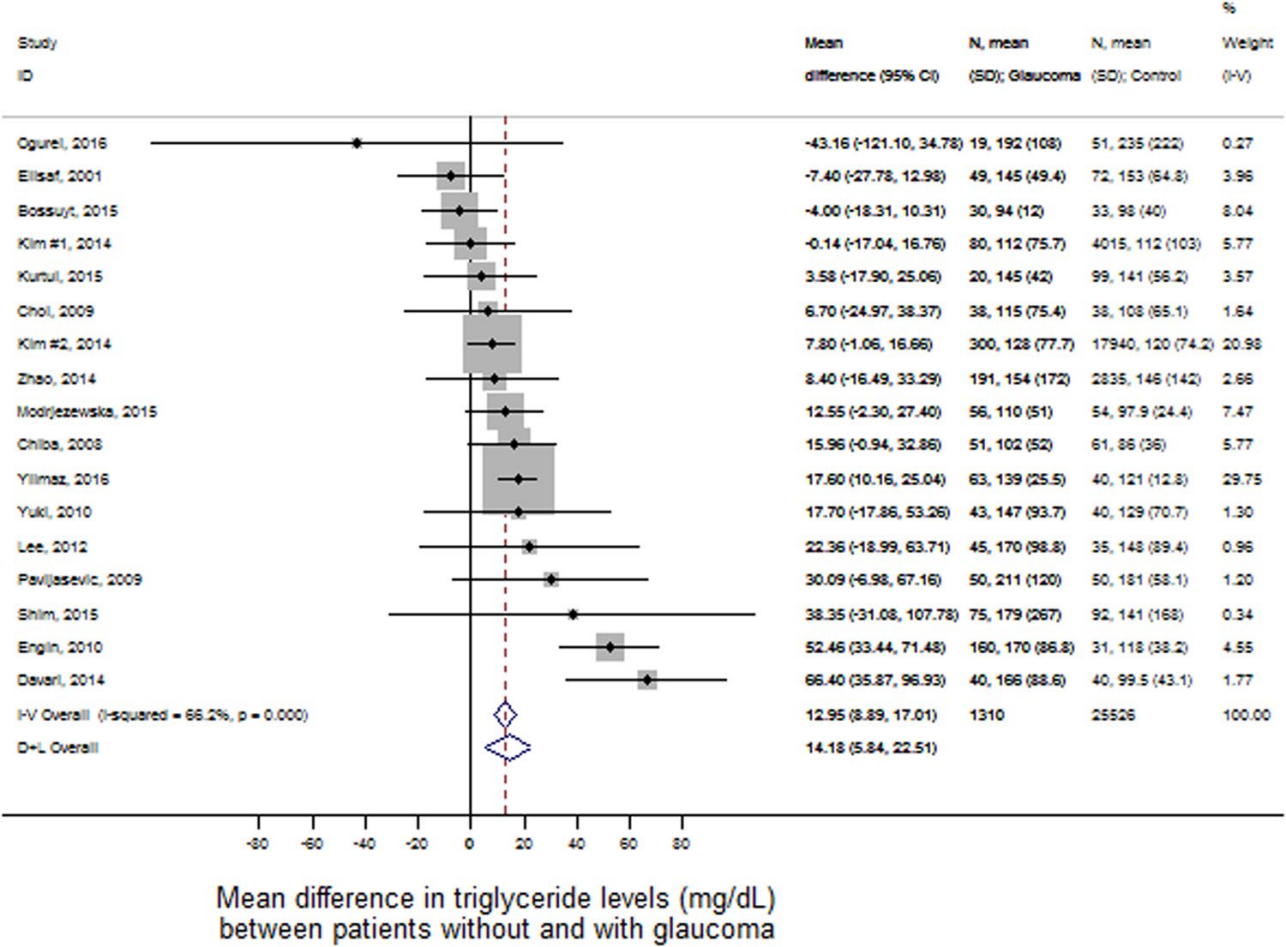
Random-effects meta-analysis of triglyceride level differences between patients with glaucoma and controls. The differences are expressed on an absolute scale, i.e. they represent differences in mg/dL. “I-V Overall” represents the fixed effects pooled estimate, and “D+L Overall” the random-effects pooled estimate. Grey boxed surrounding the individual study estimates are proportional to the fixed effects weight of the study. Diamonds surrounding the pooled estimates represent 95% confidence intervals. Abbreviations: 95%CI - 95% confidence interval, SD - Standard Deviation﻿.

**Figure 2:**
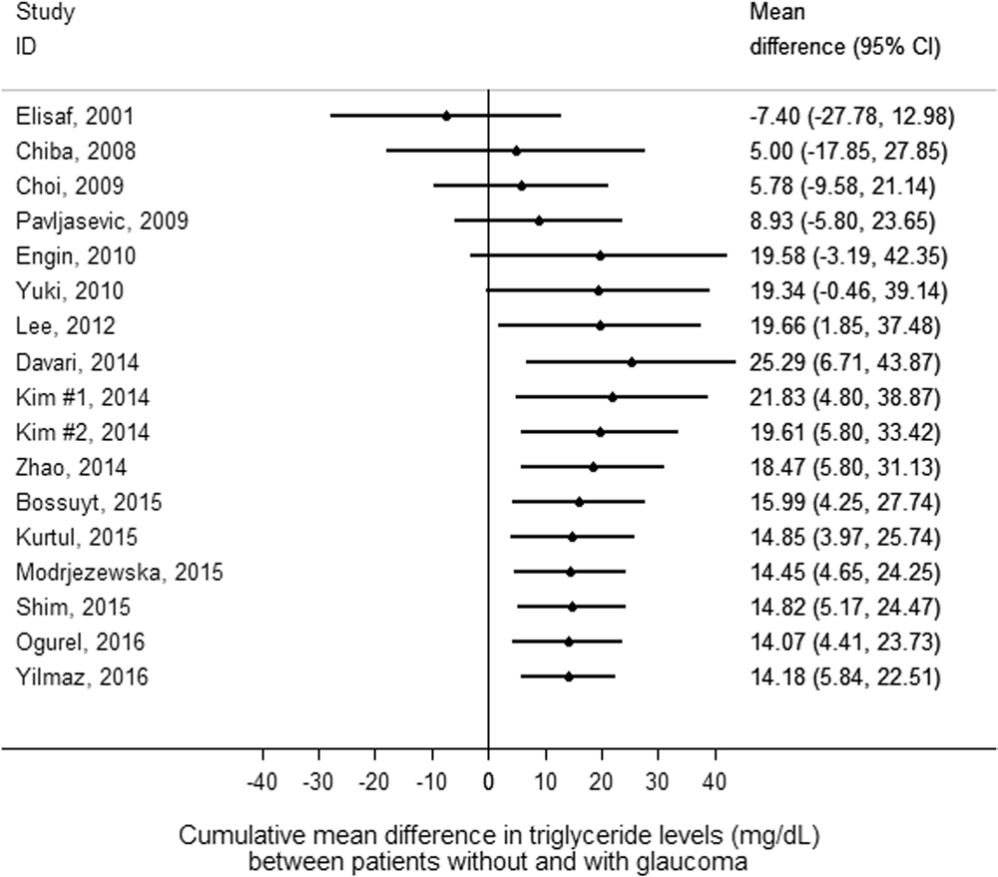
Cumulative meta-analysis of triglyceride level differences between patients with glaucoma and controls. Studies are sorted by calendar time. The estimates represent cumulative mean absolute differences in triglyceride levels (in mg/dL) between patients with glaucoma and controls. After the study of Lee *et al*., which appeared in 2012, all cumulative difference estimates indicate a statistically significantly higher average triglyceride level in glaucoma patients.

Patients with glaucoma had higher mean levels of triglycerides than patients without glaucoma. In detail, the pooled mean absolute difference in triglycerides between patients with and without glaucoma was 14.2 mg/dL in random-effects analysis (95%CI: 5.8–22.5, p < 0.0001, Fig. 1), and 13.0 mg/dL in fixed-effects analysis (95%CI: 8.9–17.0, p < 0.0001, Fig. 1). Upon inspection of study weights (gray boxes in Fig. 1), we observed that only 2 studies (Kim *et al*. #2. 2014, and Yilmaz *et al*. 2016) contributed half of the information to the pooled difference in triglycerides. On a standardized mean difference (SMD) scale, patients with glaucoma had a 0.21 standard deviations (SD) higher triglyceride level than patients without glaucoma (95%CI: 0.09–0.34, p < 0.0001, Supplementary Figure 2). The corresponding fixed-effects estimate was 0.15 SD (95%CI: 0.08–0.22, p < 0.0001). In cumulative meta-analysis, we found that significantly higher mean triglyceride levels were consistently found after the study of Lee *et al*., which appeared in 2012 (Fig. 2).

**Figure 3:**
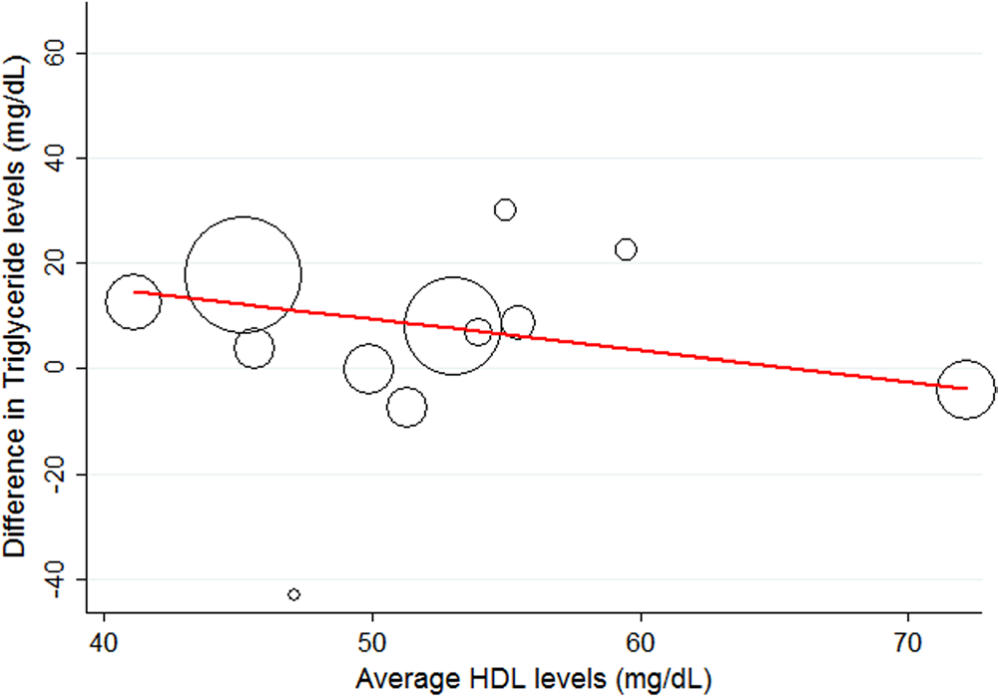
Random-effects meta-regression of triglyceride level differences between patients with glaucoma and controls according to within-study mean high-density-lipoprotein (HDL) levels. The red line represents a line of best fit from meta-regression. The diameter of the hollow circles reflects the weight of the individual studies in the random-effects meta-regression. The slope of the line of best fit declines with increasing HDL levels, which suggests that differences in triglyeride levels between glaucoma patients and controls are strongest in patient populations with low average HDL levels.

In both meta-analyses of absolute and standardized mean triglyceride differences between patients with glaucoma and controls, we observed statistically significant evidence for heterogeneity (I^2^ for the absolute difference analysis = 66.2%, heterogeneity χ^2^ = 47.4 on 16 degrees of freedom, p < 0.0001). This finding suggests that random effects estimates may be more appropriate than fixed effects estimates. Further, this relative large heterogeneity estimate is consistent with the assumption that other between-study differences may at least partly explain the overall difference in triglyceride levels between glaucoma patients and controls. To explore these potential factors, we performed a meta-regression (Table 3). In detail, we investigated the covariables age, sex, diabetes, BMI, LDL cholesterol, HDL cholesterol, total cholesterol, systolic blood pressure, diastolic blood pressure, and mean within-study triglyceride levels. None of these variables were significantly associated with the difference in triglycerides between patients with and without glaucoma, except for HDL, which was “borderline statistically significantly” associated with the triglyceride difference. However, considering that (1) meta regression has a comparably low power, (2) some covariables (e.g. diabetes, n = 5 studies) were not completely observed for all 17 studies (Tables 1, 2 and 3), and (3) the use of lipid-lowering medications was not documented in most studies (Table 2), we focussed our interpretation of meta-regression results on reductions in I^2^ upon adding the covariables. Here, our analysis suggested that study-level HDL cholesterol was an important modifier of the glaucoma-triglyceride-association. In detail, a 10 mg/dL increase in HDL cholesterol was associated with a 6 mg/dL reduction in the triglyceride difference between patients with and without glaucoma (Difference = −5.92, 95%CI: −12.55–0.71, p = 0.075, Fig. 3), which implies that studies with higher mean HDL cholesterol levels generally showed smaller difference in triglycerides between patients with and without glaucoma. Further evidence for this hypothesis comes from I^2^ estimates. In detail, addition of HDL cholesterol reduced the amount of unexplained variation in the triglyceride-glaucoma association from 30.4% to 4.2%. Diabetes may be another modifier of the triglyceride-glaucoma association, with meta-regression suggesting a higher contribution of triglycerides to glaucoma in patients that were diabetic. However, due to the small number of studies with observed diabetes data (n = 5), these results remain hypothesis-generating. In terms of within-study average triglyceride levels, we did not observe evidence for an association with triglyceride difference between glaucoma patients and controls. Studies with higher mean triglyceride levels did not appear to have a greater difference between glaucoma patients and controls than studies with lower mean triglyceride levels. Otherwise, the difference in triglycerides between patients with and without glaucoma prevailed in almost all meta-regressions (Table 3), which is consistent with the concept that elevated triglycerides may be associated with glaucoma independent of covariables such as age, sex, BMI and total cholesterol.

**Table 3:** Meta regression models exploring potential determinants of the difference in triglyceride levels between patients with and without glaucoma.

Model	N of studies	Variable	Estimate of triglyceride difference (mg/dL)	95%CI	p	Base model I^2^	Residual I^2^
Base Model	17	Constant*	14.48	3.97–24.98	0.010	66.3%	N/A
Model #1	17	Age (per 5 years increase above 60)	−0.54	−6.24–5.17	0.843	66.3%	68.3%
		Constant*	14.26	2.82–25.71	0.018		
Model #2	16	Female sex (per 10% increase in prevalence above 50% prevalence)	−0.76	−9.89–8.36	0.860	68.4%	70.4%
		Constant*	15.23	2.35–28.12	0.024		
Model #3	5	Diabetes (per 10% increase in prevalence of 15% prevalence)	8.74	−17.34–34.82	0.364	51.8%	37.6%
		Constant*	10.00	−8.48–28.48	0.184		
Model #4	10	BMI (per 1 kg/m² increase above 25 kg/m²)	−0.33	−4.65–3.99	0.866	45.7%	43.0%
		Constant*	6.92	−2.72–16.56	0.137		
Model #5	9	LDL cholesterol (per 10 mg/dL increase above 120 mg/dL)	−0.20	−7.19–6.79	0.948	43.3%	50.3%
		Constant*	7.79	−4.76–20.35	0.186		
Model #6	12	HDL cholesterol (per 10 mg/dL increase above 50 mg/dL)	−5.92	−12.55–0.71	0.075	30.4%	4.2%
		Constant*	9.30	3.69–14.91	0.004		
Model #7	15	Total cholesterol (per 10 mg/dL increase above 200 mg/dL)	−2.32	−9.00–4.37	0.468	69.3%	70.6%
		Constant*	16.33	3.55–29.12	0.016		
Model #8	7	Systolic blood pressure (per 10 mmHg increase above 125 mmHg)	−5.89	−26.15–14.37	0.489	0.0%	0.0%
		Constant*	6.95	−4.12–18.03	0.167		
Model #9	7	Diastolic blood pressure (per 10 mmHg increase above 75 mmHg)	−4.80	−30.98–21.38	0.657	0.0%	0.0%
		Constant*	9.78	0.34–19.22	0.045		
Model #10	17	Mean within-study triglyceride level (per 10 mg/dL increase above 130 mg/dL)	1.72	−2.15–5.59	0.358	66.3%	62.6%
		Constant*	14.34	3.84–24.85	0.011		

Covariables were centred at or close to their mean across studies.

## Discussion

In this meta-analysis of 1 391 patients with glaucoma compared to 25 575 without glaucoma included in 17 cross-sectional studies, we found higher mean triglyceride levels in patients with glaucoma as compared to controls. The pooled mean absolute difference in triglycerides between patients with and without glaucoma was 14.2 mg/dL.

The association of triglycerides and glaucoma has so far been a matter of ongoing debate. Some studies found a significant association^9, 10^, while other studies could not confirm this association (Table 1)^13, 14^. These different results are reflected by the considerable amount of heterogeneity found in this meta-analysis. One reason for this heterogeneity may be that different types of glaucoma were included in this meta-analysis (Table 2). Furthermore, inclusion criteria were different among the included studies. For example visual field defects had to be present in some but not all studies. Three studies had no definition for who was considered to be a patient with glaucoma^16–18^ and in one study patients were simply surveyed on whether they had a glaucoma^19^. The control groups were equally dissimilar. Six studies included hospital controls, four community controls and seven did not report on control group recruitment. Nine studies gave no definition of the inclusion criteria for the control group. Likewise, exclusion criteria were different in the included studies. This may be particularly relevant for a modifying influence of co-medications on triglyceride levels. For example, patients using lipid-lowering medication were excluded in six of the seventeen studies and included in one study, while the other ten studies did not report any data on the use of lipid-lowering medication. Moreover, diabetes mellitus, smoking and cardiovascular disease was included in some studies, excluded in others, or not reported on (Table 2).

However, not only co-medications may modify the association of triglycerides and glaucoma, but also medications used for glaucoma treatment. Importantly, topical beta-blockers, which are a cornerstone treatment for glaucoma, have been shown to increase systemic triglyceride levels in a study on 28 healthy volunteers taking topical timolol^20^. Given the fact that it is very likely that the patients in this meta-analysis had a high prevalence of topical beta-blocker use, this may have increased the difference in triglyceride levels between patients and controls. Therefore, future studies should address this potential bias.

In meta-regression, we found that HDL cholesterol was a modifier of the glaucoma-triglyceride-association. Specifically, studies including patients with lower average HDL cholesterol levels showed larger differences in triglyceride levels between patients with and without glaucoma. As HDL levels are known to inversely correlate with triglyceride levels suggesting a dynamic interaction^4, 21^, it seems rather unlikely that HDL itself is a modifier of the glaucoma-triglyceride-association. We hypothesize that lipid-lowering interventions may cause this surprising result. In a recent meta-analysis, statin use was associated with a reduced incidence of glaucoma^22^. Life-style interventions influencing the metabolism of HDL such as smoking, weight loss and physical activity, and the use of lipid-lowering medications are poorly reported in most studies. For example the percentage of patients using lipid-lowering medications was given in only one study^23^. Therefore we could not account for lipid-lowering interventions in this meta-regression.

Likewise, triglycerides appeared to have a higher contribution to glaucoma in patients with diabetes mellitus, a known risk factor for glaucoma^24, 25^. Ko *et al*.^15^ found that diabetes was significantly associated with glaucoma only if triglycerides were excluded from the multivariable regression model. High triglyceride levels seem to be associated with worse glycaemic control^26^. Zhao *et al*.^19^ could show that HbA1c levels are associated with glaucoma. Our findings of diabetes mellitus as a modifier may therefore reflect the association of glaucoma with worse glycaemic control.

In this meta-analysis triglyceride levels were significantly higher in patients with glaucoma in the random-effects analysis. A recent study by Ko^15^ *et al*. found a significant inverse correlation between high triglyceride levels and glaucoma. As no actual triglyceride levels are given by Ko^15^ *et al*., it was not included in this meta-analysis. They used a rather high cut-off at ≥200 mg/dL. As the difference in triglyceride levels in this meta-analysis was 14.2 mg/dL, differences in the percentage of high triglycerides levels using a cut-off at ≥200 mg/dL would probably not be found and this may explain this discrepancy. Further no information on the use of lipid-lowering drugs, especially fibrates, is given, which might also explain the inverse correlation.

Elevated IOP does not seem sufficient to solely explain the pathogenesis of glaucoma^1^. Other risk factors, especially the dysfunction of the vasculature supplying the optic nerve and the surrounding tissue, have therefore been implicated^3^. Triglycerides are an independent risk factor for cardiovascular disease^27^, although their role in the pathogenesis of atherosclerosis is controversial^28^. Patients diagnosed with glaucoma have been shown to have an increased risk of cardiovascular mortality^29, 30^, which is primarily explained by common risk factors such as hypertriglyceridemia^31, 32^. Glaucoma medication has also been suggested to influence lipid levels, however evidence is very limited^33, 34^.

Finally, we want to mention three limitations of the study. First, the current analysis only incorporates observational data. Therefore, we want to stress that although our study provides strong statistical evidence that triglycerides are associated with glaucoma, we cannot prove causality. Specifically, we cannot answer whether hypertriglyceridemia leads to glaucoma via a potential effect on intraocular pressure, or whether triglycerides may have a pathogenic impact on glaucoma independently of intraocular pressure. Second, the observational nature of the included studies does not allow us to evaluate whether interventions that lower triglyceride levels can modify glaucoma development and/or outcomes. Randomised clinical studies are needed to investigate whether the use of triglyceride-lowering drugs may be useful in the prevention or treatment of glaucoma. Preliminary evidence supporting a role for lipid-lowering drugs towards glaucoma outcomes stems from a large British registry study, in which statin use was associated with lower IOP^35^. This is relevant also for our meta-analysis, given the fact that statins not only lower LDL cholesterol but also triglyceride levels, which may in turn ameliorate the adverse association of triglycerides with glaucoma. Although the impact of statins on IOP did not prevail after adjusting for beta-blockers in the aforementioned study, future studies should clearly explore how lipid-lowering drugs affect triglyceride levels and their role on glaucoma development and progression. Third, we obtained convincing statistical evidence that patients with glaucoma have higher average triglyceride levels than controls. However, it remains to be investigated whether this statistically significant absolute difference of approximately 14 mg/dL is not only statistically significant but also clinically relevant. This question could be addressed by future studies, which employ a prospective design looking at clinical outcomes of glaucoma pathogenesis or glaucoma progression.

In conclusion, our meta-analysis summarizes a large body of evidence from case-control studies on the glaucoma-triglyceride association. The observed results are consistent with the concept that hypertriglyceridemia may contribute to glaucoma pathogenesis, and support the future conduct of randomized trials on triglyceride-lowering interventions for prevention or treatment of glaucoma.

## Methods

### Search history

From July 12^th^, 2014, until August 2^nd^, 2016, we used an Ovid Interface to search for the following medical subject headings in the databases PubMed, EMBASE, and The Cochrane Library: “glaucoma” AND “triglycerides”; “intraocular pressure” AND “triglycerides”; “glaucoma” AND “dyslipidemia”; and “intraocular pressure” AND “dyslipidemia”. The reference lists of included studies were additionally scanned to identify potentially relevant reports. Two investigators (LP & FP) performed the literature search and study selection.

**Figure 4:**
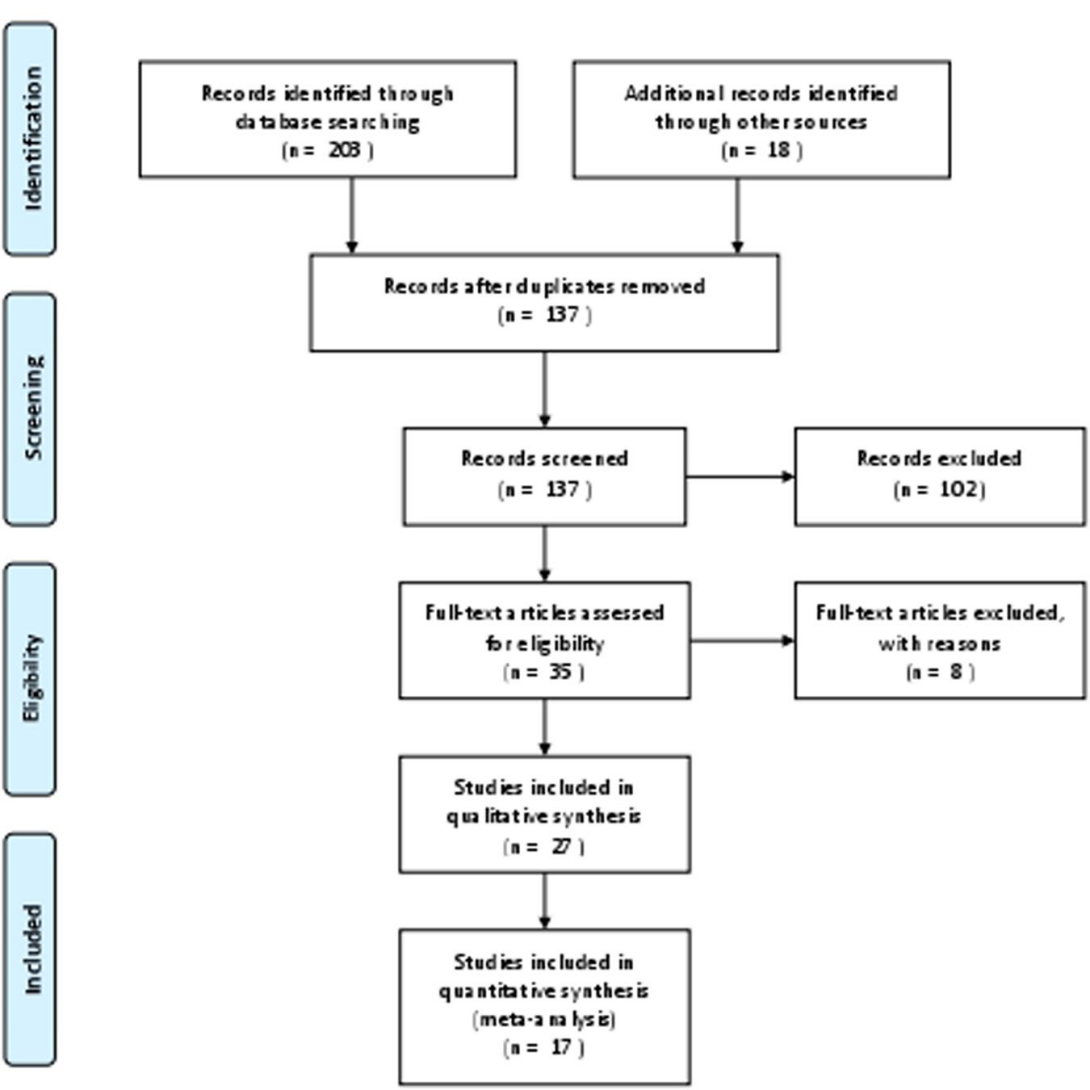
Prisma Flow Diagram of the study selection process. Adapted from: Moher D, Liberati A, Tetzlaff J, Altman DG, The PRISMA Group (2009). Preferred Reporting Items for Systematic Reviews and Meta-Analyses: The PRISMA Statement.PLoS Med 6(7): e1000097. doi:10.1371/journal.pmed1000097. For more information, visit http://www.prisma-statement.org.

Studies were included if they reported the triglyceride levels in patients with glaucoma and compared to control patients. Only studies in English language were included. All abstracts and conference proceedings that are not published in peer-reviewed journals were excluded. If original data or exact numbers were not available, they were not included in the quantitative analysis (Fig. 4).

### Statistical methods

All statistical analyses were performed using Stata (Windows version 14.0, Stata Corp., Houston, TX, USA). Differences in mean triglyceride levels between patients with and without glaucoma were analysed using random- and fixed-effects meta-analysis (Stata routine metan). Differences were analysed both on an absolute scale (i.e. difference in triglycerides between patients with and without glaucoma in mg/dL) and on a standardized scale (i.e. difference in triglycerides between the two groups in units of standard deviation (so-called standardized mean differences)). The I^2^ statistic was evaluated for quantifying the extent of unexplained variation in the triglyceride-glaucoma association (i.e. heterogeneity). The user-contributed suite metacum was applied for cumulative meta-analysis of absolute differences in triglyceride levels between patients with and without glaucoma. Meta-regressions were performed on the absolute scale with Stata’s user-contributed routine metareg in order to explore potential modifiers of the triglyceride-glaucoma association. The full analysis code is available on request from FP.

## Electronic supplementary material

Supplementary Figures
