# Supplementary material for: Triglycerides and Open Angle Glaucoma – A Meta-analysis with meta-regression
Source: Sci Rep. 2017 Aug 10;7:7829. doi: 10.1038/s41598-017-08295-1 (PMC5552857; doi:10.1038/s41598-017-08295-1)
Supplement: Supplementary file 1 — Supplementary Figures [file 41598_2017_8295_MOESM1_ESM.pdf]

Supplementary tables and figures

Triglycerides and Open Angle Glaucoma – A Meta-analysis with meta-regression

A meta-analysis and meta-regression of 17 case-control studies on the association of triglycerides with glaucoma

Laura Pertl[1], Georg Mossböck[1], Andreas Wedrich[1], Martin Weger[1], Oliver Königsbrügge[2], Günther Silbernagel[3], and Florian Posch[4]

1. Department of Ophthalmology
   Medical University of Graz
   Auenbruggerplatz 4
   8036 Graz, Austria

2. Clinical Division of Haematology and Haemostaseology
   Department of Internal Medicine I
   Medical University of Vienna
   Währinger Gürtel 18-20
   1090 Vienna, Austria

3. Division of Angiology
   Department of Internal Medicine
   Medical University of Graz
   Auenbruggerplatz 15
   8036 Graz, Austria

4. Division of Oncology
   Department of Internal Medicine
   Medical University of Graz
   Auenbruggerplatz 15
   8036 Graz, Austria

Supplementary Figure 1

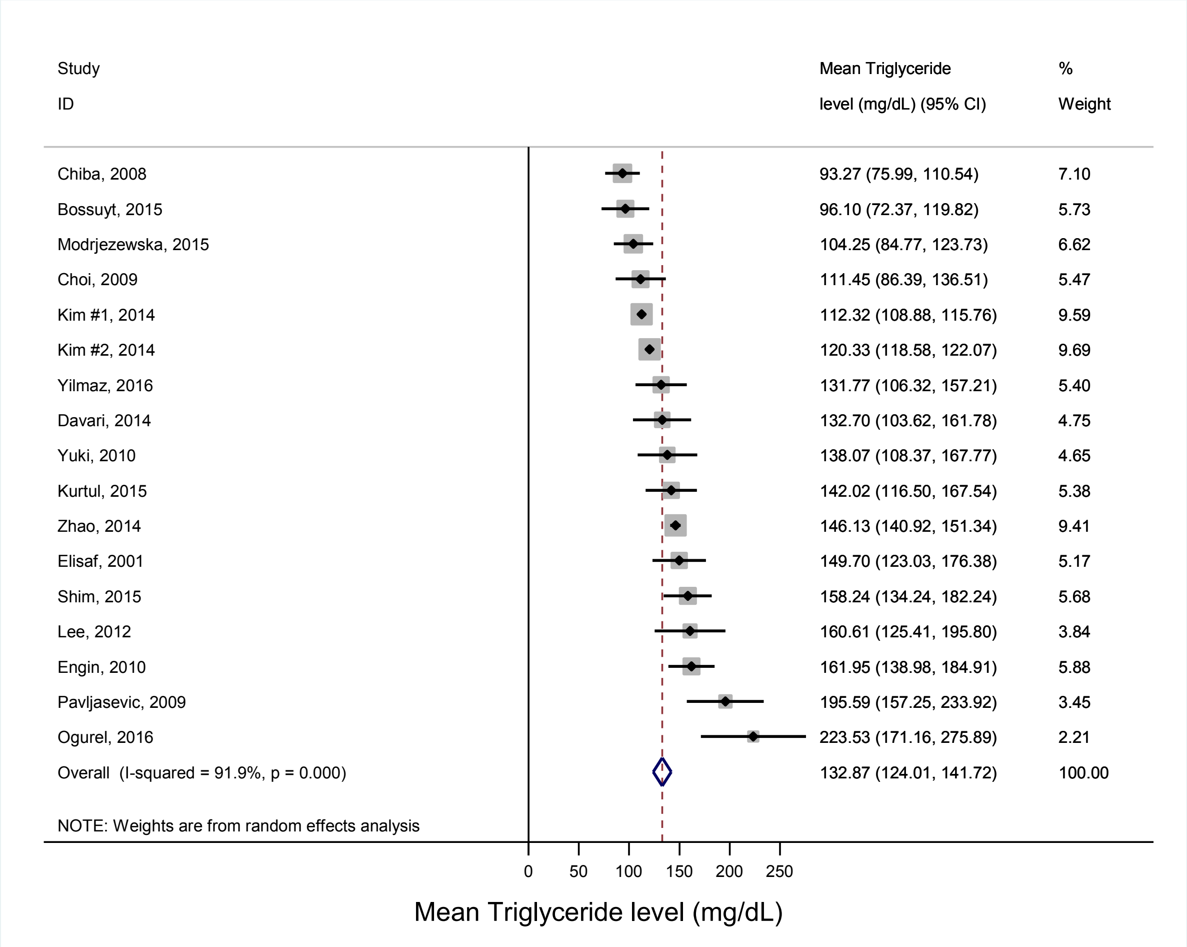

Supplementary Figure 2

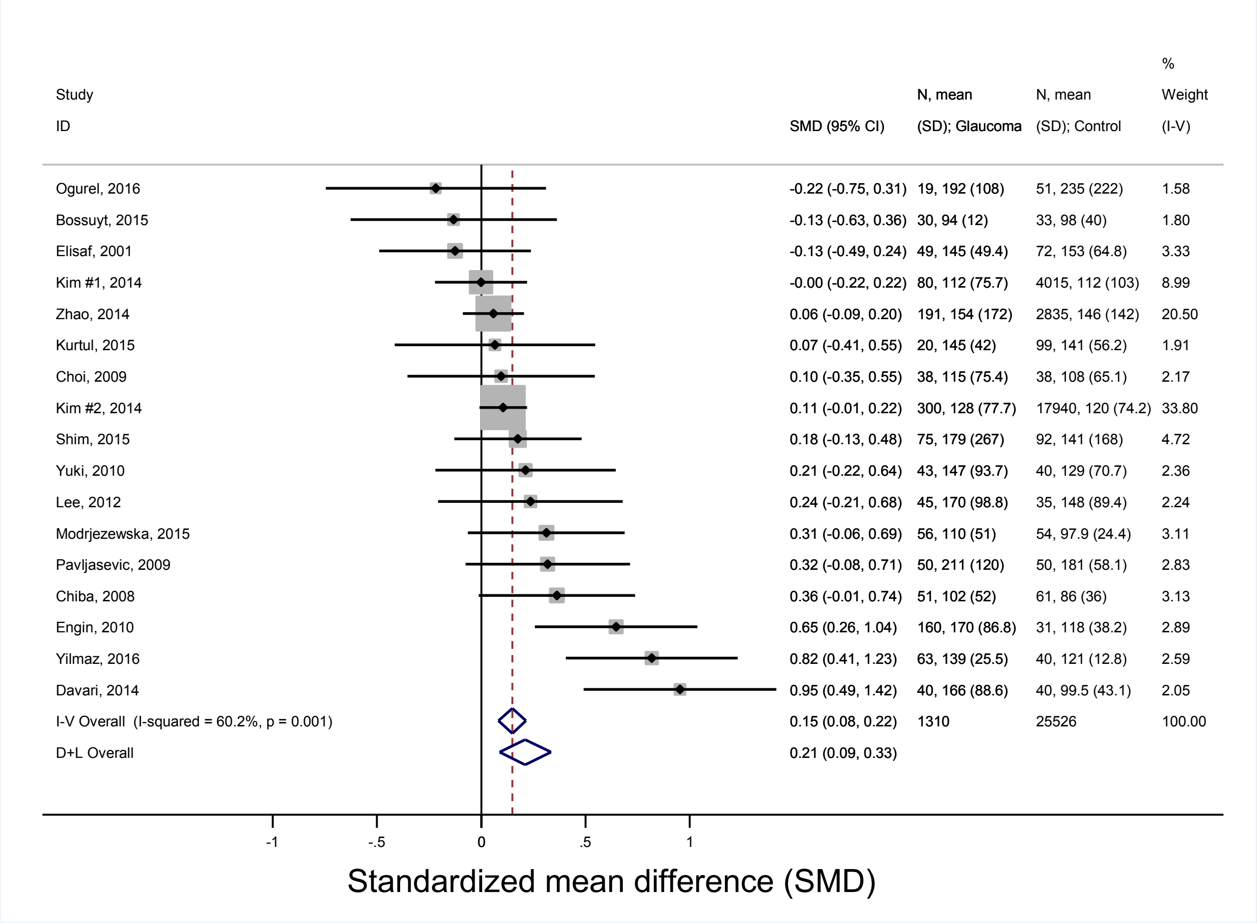

Supplementary Figure 3

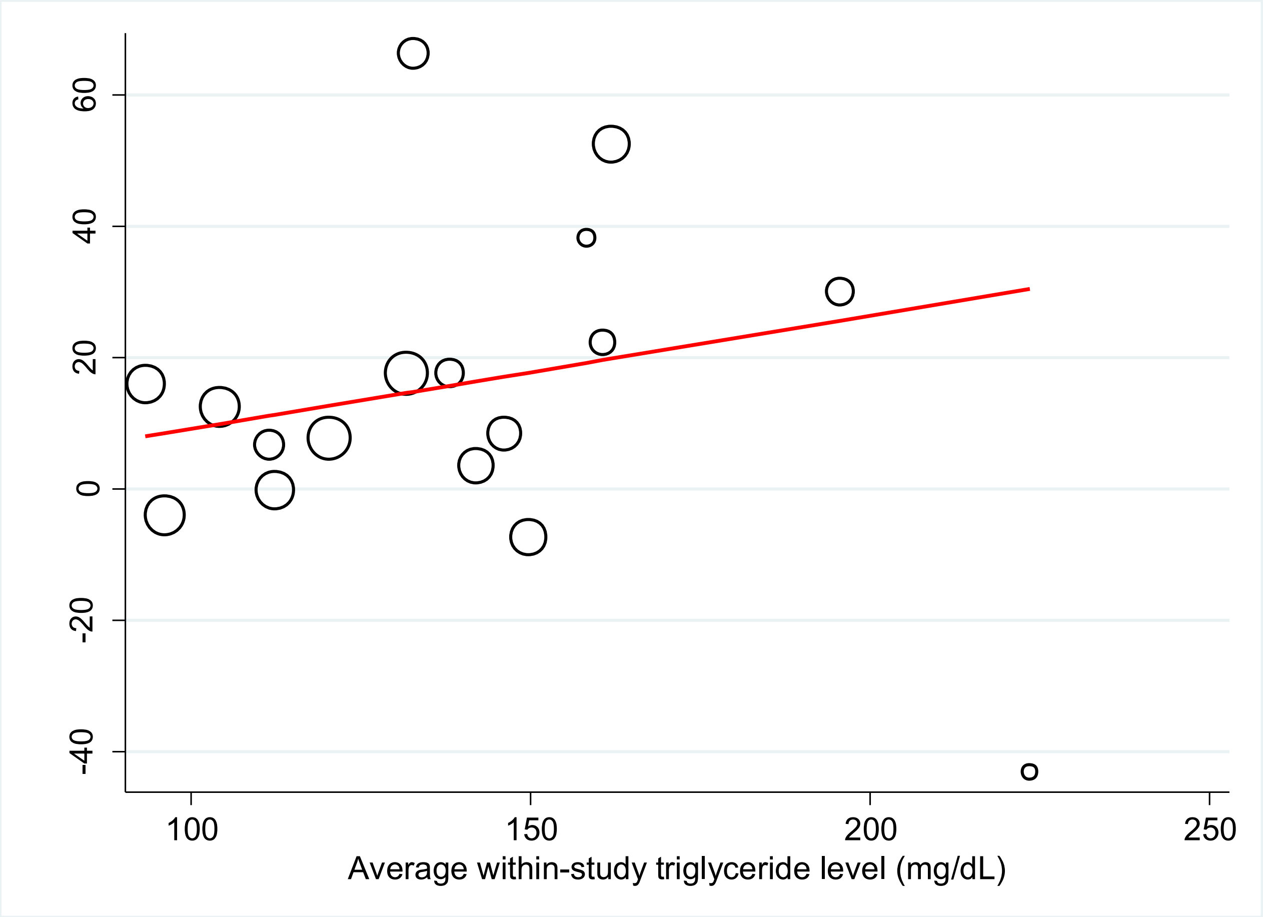

Supplementary figure legends

Supplementary Figure 1. Random-effects meta-analysis of mean triglyceride levels. The within-study mean triglyceride levels show a large degree of heterogeneity, with mean levels ranging from 93mg/dL to 224 mg/dL.

Supplementary Figure 2. Random-effects meta-analysis of triglyceride level differences between patients with glaucoma and controls. The differences are expressed on a standardized scale, i.e. they represent standardized mean differences (SMDs). The unit of these SMDs is 1 standard deviation.

Supplementary Figure 3. Random-effects meta-regression of triglyceride level differences between patients with glaucoma and controls according to within-study mean triglyceride levels. The red line represents a line of best fit from meta-regression. The diameter of the hollow circle reflects the weight of the individual studies in the random-effects meta-regression.
